# 1,25(OH)_2_D_3_ and dexamethasone additively suppress synovial fibroblast activation by CCR6^+^ T helper memory cells and enhance the effect of tumor necrosis factor alpha blockade

**DOI:** 10.1186/s13075-018-1706-9

**Published:** 2018-09-20

**Authors:** Wendy Dankers, Claudia González-Leal, Nadine Davelaar, Patrick S. Asmawidjaja, Adriana M. C. Mus, Johanna M. W. Hazes, Edgar M. Colin, Erik Lubberts

**Affiliations:** 1 Department of Rheumatology, Erasmus MC, Rotterdam, the Netherlands; 2 Department of Immunology, Erasmus MC, Rotterdam, the Netherlands; 3 Department of Internal Medicine, Erasmus MC, Rotterdam, the Netherlands; 4 Erasmus MC University Medical Center, Wytemaweg 80, 3015CN Rotterdam, The Netherlands

**Keywords:** Rheumatoid arthritis, Th17, Vitamin D, Dexamethasone, CCR6

## Abstract

**Background:**

Despite recent improvements in the treatment of rheumatoid arthritis (RA), an insufficient treatment response and the development of treatment resistance in many patients illustrates the need for new therapeutic strategies. Chronic synovial inflammation could be suppressed by targeting RA synovial fibroblast (RASF) activation by, for example, interleukin (IL)-17A-producing CCR6^+^ T helper memory (memTh) cells. Here, we modulated this interaction by combining the active vitamin D metabolite 1,25(OH)_2_D_3_ with dexamethasone (DEX) and explored the potential therapeutic applications.

**Methods:**

CCR6^+^ memTh cells from peripheral blood mononuclear cells (PBMCs) of healthy donors or treatment-naive early RA patients were cultured alone or with RASF from established RA patients for 3 days and treated with or without 1,25(OH)_2_D_3_, DEX, or etanercept. Treatment effects were assessed using enzyme-linked immunosorbent assay (ELISA) and flow cytometry.

**Results:**

1,25(OH)_2_D_3_, and to lesser extent DEX, reduced production of the pro-inflammatory cytokines IL-17A, IL-22, and interferon (IFN)γ in CCR6^+^ memTh cells. Tumor necrosis factor (TNF)α was only inhibited by the combination of 1,25(OH)_2_D_3_ and DEX. In contrast, DEX was the strongest inhibitor of IL-6, IL-8, and tissue-destructive enzymes in RASF. As a result, 1,25(OH)_2_D_3_ and DEX additively inhibited inflammatory mediators in CCR6^+^ memTh-RASF cocultures. Interestingly, low doses of mainly DEX, but also 1,25(OH)_2_D_3_, combined with etanercept better suppressed synovial inflammation in this coculture model compared with etanercept alone.

**Conclusion:**

This study suggests that 1,25(OH)_2_D_3_ and DEX additively inhibit synovial inflammation through targeting predominantly CCR6^+^ memTh cells and RASF, respectively. Furthermore, low doses of DEX and 1,25(OH)_2_D_3_ enhance the effect of TNFα blockade in inhibiting RASF activation, thus providing a basis to improve RA treatment.

**Electronic supplementary material:**

The online version of this article (10.1186/s13075-018-1706-9) contains supplementary material, which is available to authorized users.

## Background

Rheumatoid arthritis (RA) is a chronic inflammatory autoimmune disease characterized by inflamed synovial joints resulting in pain, fatigue, and disability in patients. Although treatment has improved over the last decades, many patients do not reach clinical remission, show progressive joint damage, or become resistant to their treatment [1]. Furthermore, the current therapies include the use of expensive biological disease-modifying antirheumatic drugs (DMARDs) which pose a burden on the healthcare budget. Therefore, there is still a need to find new therapeutic options and to improve currently available treatments.

The main therapeutic goal in RA is to stop the chronic synovial inflammation and thereby prevent the subsequent cartilage and bone damage in the affected joint. Synovial fibroblasts play an important role in this process since they can secrete proinflammatory cytokines that attract and activate immune cells, produce tissue-destructive enzymes, and invade cartilage [2, 3]. How these RA synovial fibroblasts (RASF) are activated is currently unknown, although one of the hypotheses is that they are activated by infiltrating immune cells. Previously we have shown that CCR6^+^, but not CCR6^–^, memory T helper (memTh) cells can activate RASF [4].

CCR6^+^ memTh cells are characterized by interleukin (IL)-17A production and RAR-related orphan C receptor (RORC) expression. This subset contains the classic Th17 cells, but also contains, for example, Th17.1 cells that produce high levels of interferon (IFN)γ [5]. Other evidence that suggests a role for these IL-17A-producing memTh cells is that Th17 cells and IL-17-mediated signaling are required for the development of murine autoimmune arthritis [6, 7]. Furthermore, CCR6^+^ memTh cells are more activated and more prevalent in the blood of treatment-naive early RA patients and are also found in the synovial fluid of RA patients [4, 8].

Upon interaction with CCR6^+^ memTh cells, RASF secrete proinflammatory mediators such as IL-6, matrix metalloproteases (MMPs), and prostaglandin E2 (PGE2) via IL-17A and tumor necrosis factor (TNF)α. In turn, these molecules, especially PGE2, further activate the CCR6^+^ memTh cells to produce more IL-17A, thereby creating a proinflammatory feedback loop that could drive the chronic synovial inflammation [4, 9]. Therefore, inhibiting this proinflammatory loop may be beneficial in the treatment of RA.

We have previously shown that combining the active vitamin D metabolite 1,25(OH)_2_D_3_ with TNFα blockade, a commonly used therapy in RA, additively suppresses the proinflammatory loop between CCR6^+^ memTh and RASF [10]. However, clinical translation of these findings is challenging since high concentrations of 1,25(OH)_2_D_3_ were used. Interestingly, the effects of 1,25(OH)_2_D_3_ on Th17-related cytokines, such as IL-17A, TNFα, and IL-22, can be augmented through combination with dexamethasone (DEX) [11]. DEX is a synthetic glucocorticoid (GC) which is clinically used for fast resolution of inflammation and is often combined with vitamin D supplements to prevent the osteoporotic side effects of the drug [12].

Given the immunomodulatory capacities of both DEX and 1,25(OH)_2_D_3_, we here investigated whether this combination could suppress CCR6^+^ memTh cells, RASF, and their interaction. Furthermore, the potential use of these findings for improving current anti-TNFα therapy is explored.

## Methods

### Subjects

Healthy control peripheral blood mononuclear cells (PBMCs) were obtained from buffycoats (Sanquin, Amsterdam, the Netherlands). For validation of findings in healthy PBMCs, RA PBMCs were isolated from treatment-naive RA patients who were embedded in the tREACH study, which was ethically approved by the METC Rotterdam. Relevant clinical information is summarized in Additional file 1 (Table S1). RASF were grown from synovial explants after joint replacement surgery. All patients signed informed consent.

### Cell sorting

PBMCs were isolated from peripheral blood using ficoll-based cell separation and frozen in liquid nitrogen until use. For sorting of CCR6^+^ memTh cells (CD4^+^CD45RO^+^CCR6^+^CD25^low/int^), PBMCs were stained using antibodies against CD4, CCR6, CD25 (BioLegend, San Diego, CA, USA), and CD45RO (BD Biosciences, San Diego, CA, USA). Before sorting CCR6^+^ memTh cells, the cells were prepurified using CD4 microbeads via automated magnetic-activated cell sorting (Miltenyi Biotec, Leiden, The Netherlands). Dead cells were excluded using 4′6-diamidino-2-phenylindole dilactate (DAPI) and CCR6^+^ memTh cells were sorted on a FACSAriaIII sorter (BD Biosciences).

### Cell culture

RASF were obtained by culturing small synovial biopsies in a culture flask with Dulbecco’s modified Eagle’s medium (DMEM; Gibco, Waltham, MA, USA), supplemented with 10% fetal calf serum (FCS; Gibco) and 100 IU/ml penicillin/streptomycin (pen/strep; Lonza, Verviers, Belgium). After RASF were grown out of the synovial biopsies, cells were passaged and used for experiments between passage 3 and 8. For coculture with sorted T cells or stimulation experiments, RASF were plated at a density of 1 × 10^4^ cells/well in a 96-well plate 24 h before the T cells or stimulation medium were added. Where indicated, RASF were stimulated with 2 ng/ml recombinant TNFα and 5 ng/ml recombinant IL-17A (R&D Systems, Minneapolis, MN, USA).

Sorted CCR6^+^ memTh cells were stimulated with 300 ng/ml soluble anti-CD3 and 400 ng/ml soluble anti-CD28 (Sanquin, Amsterdam, the Netherlands) at a density of 2.5 × 10^4^ cells/ml in Iscove’s modified Dulbecco’s medium (IMDM; Gibco) supplemented with 10% FCS, 100 IU/ml pen/strep, 2 mM l-glutamine, and 50 μM β-mercaptoethanol (Sigma-Aldrich, St. Louis, MO, USA). Cells were treated with or without 1,25(OH)_2_D_3_ (Leo Pharmaceutical Products, Ballerup, Denmark), dexamethasone (Sigma-Aldrich), and etanercept (anti-TNFα, Pfizer, New York, NY, USA) dissolved in 100% ethanol at the indicated concentrations and added for the full duration of the culture simultaneously with the stimulatory compounds. Control conditions contained an equal volume of 100% ethanol which never exceeded 0.1%.

### Flow cytometry

Cultured cells were restimulated with 50 ng/ml phorbol 12-myristate 13 acetate (PMA), 500 ng/ml ionomycin (Sigma-Aldrich), and GolgiStop (BD Biosciences) for 4 h. Cells were then stained with Fixable Viability Dye eFluor506 (eBioscience, San Diego, CA, USA), fixated with 2% paraformaldehyde and permeabilized using 0.5% saponine. Intracellular cytokines were stained with monoclonal antibodies against IL-17A, IL-22 (eBioscience), and IFNγ (BioLegend).

Apoptosis was assessed using 7AAD/Annexin V staining, performed according to the manufacturer’s instructions (eBioscience). All samples were measured on the FACSCantoII Flow Cytometer (BD Biosciences).

### Enzyme-linked immunosorbent assay (ELISA)

In culture supernatant after 3 days of culture, the concentration of IL-17A, IL-22, IFNγ, TNFα, IL-10, IL-6, IL-8 (Ready-Set-Go, eBioscience), MMP1, MMP3 (DuoSet ELISA, R&D Systems), and PGE2 (Prostaglandin E2 Parameter Assay Kit, R&D Systems) were measured using ELISA. The manufacturers’ protocols were followed.

### Statistical analysis

Differences between experimental treatment groups were tested using analysis of variance (ANOVA) with Bonferroni post-hoc tests. *p* values below 0.05 were considered statistically significant. Analyses were performed using Prism software version 6.01 (GraphPad Software, La Jolla, CA, USA).

## Results

### 1,25(OH)_2_D_3_-DEX combination treatment suppresses TNFα production from CCR6^+^ memTh cells

**Fig. 1:**
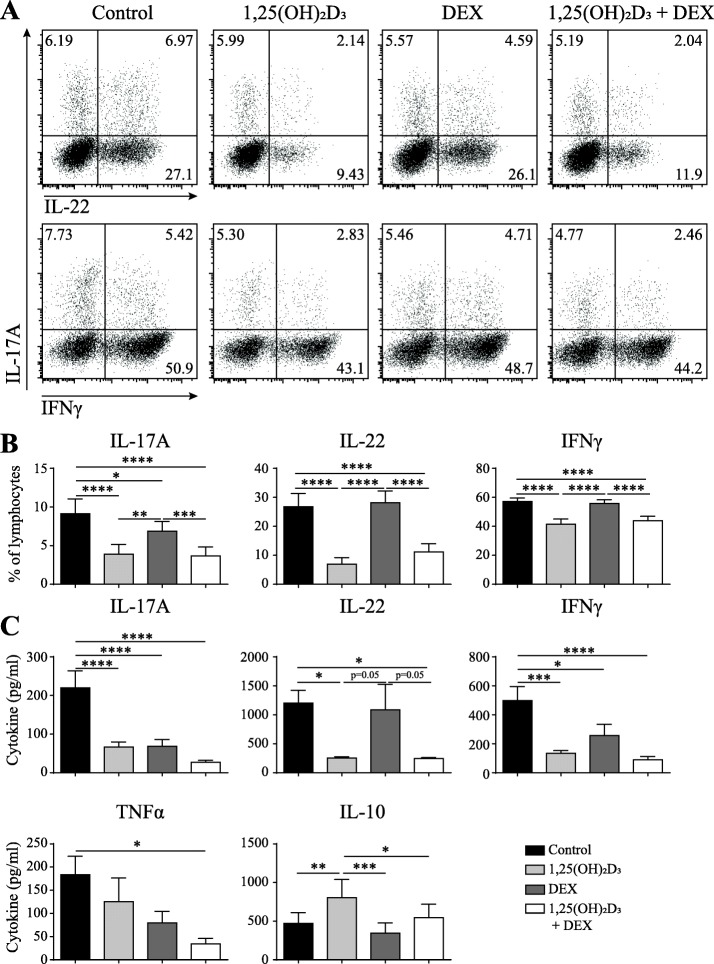
1,25(OH)_2_D_3_ and DEX additively suppress proinflammatory cytokines secreted by CCR6^+^ memTh cells. CCR6^+^ memTh cells were sorted from healthy individuals and cultured for 3 days under stimulation with anti-CD3 and anti-CD28, with or without 100 nM 1,25(OH)_2_D_3_ and 1000 nM dexamethasone (DEX). **a** Representative flow cytometry plots of interleukin (IL)-17A-, IL-22-, and interferon (IFN)γ-producing cells. **b** Quantification of flow cytometry as in **a** for six donors. **c** Cytokine production in the culture medium as measured by ELISA. Data show mean ± SEM for *n* = 6–10 healthy individuals, representative of at least three independent experiments. *p < 0.05, **p < 0.01, ***p < 0.001, ****p < 0.0001. TNF tumor necrosis factor

As a first step to examine the potential use of 1,25(OH)_2_D_3_ and DEX to inhibit synovial inflammation, CCR6^+^ memTh cells were sorted from healthy controls and cultured for 3 days with 1,25(OH)_2_D_3_, DEX, or both. In line with the previous studies, treatment with 1,25(OH)_2_D_3_ reduced the percentage of cells producing IL-17A, IL-22, and IFNγ. On the other hand, treatment with DEX did not affect the percentage of IL-22- or IFNγ-producing cells. The percentage of IL-17A-producing cells was reduced by DEX, but significantly less than by 1,25(OH)_2_D_3_. Combining 1,25(OH)_2_D_3_ and DEX had a similar effect as 1,25(OH)_2_D_3_ single treatment (Fig. 1a, b). Notably, treatment with DEX significantly reduced the amount of IL-17A and IFNγ that was produced during the 3-day culture period (Fig. 1c). Since DEX is known to induce cell death, the treatment effect on apoptosis was investigated. However, the minimal increase in apoptosis on DEX or combination exposure could not explain the difference between flow cytometry and ELISA (Additional file 1: Figure S1). Although no additive effect was observed with 1,25(OH)_2_D_3_-DEX combination treatment on IL-17A, IL-22, or IFNγ, TNFα was significantly inhibited by the combined exposure (Fig. 1c). Since 1,25(OH)_2_D_3_ can induce an anti-inflammatory phenotype in the CCR6^+^ memTh cells (Dankers et al., submitted manuscript), the combinatory effects of 1,25(OH)_2_D_3_ and DEX on IL-10 were also assessed. However, DEX did not affect IL-10 production when used alone and reduced the induction of IL-10 by 1,25(OH)_2_D_3_ when used in combination (Fig. 1c). Overall, these data show that 1,25(OH)_2_D_3_ is the strongest modulator of IL-17A, IL-22, IFNγ, and IL-10 production by CCR6^+^ memTh cells, but the combination of 1,25(OH)_2_D_3_ and DEX provides additional value through enhanced TNFα inhibition.

### 1,25(OH)_2_D_3_ and DEX additively suppress the proinflammatory feedback loop between CCR6^+^ memTh cells and RASF

**Fig. 2:**
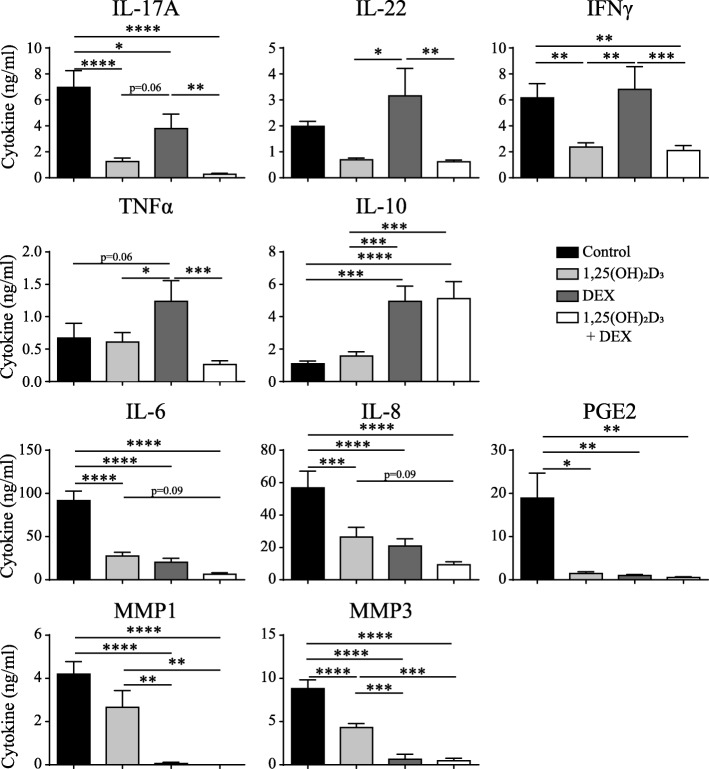
1,25(OH)_2_D_3_ and DEX additively suppress the proinflammatory feedback loop between CCR6^+^ memTh cells and RASF. CCR6^+^ memTh sorted from healthy individuals were cultured with RASF for 3 days. The cells were stimulated with anti-CD3 and anti-CD28 and treated with or without 100 nM 1,25(OH)_2_D_3_ and 1000 nM dexamethasone (DEX). After 3 days, cytokine production was measured in the culture supernatant using ELISA. Mean ± SEM are given for *n* = 10 healthy donors grown on RASF from two different donors. Data are representative of at least three independent experiments. *p < 0.05, **p < 0.01, ***p < 0.001, ****p < 0.0001. IFN interferon, IL interleukin, MMP matrix metalloprotease, PGE2 prostaglandin E2, TNF tumor necrosis factor

Since 1,25(OH)_2_D_3_ and DEX had additive effects in blocking TNFα and they were both capable of reducing the level of IL-17A that is produced by CCR6^+^ memTh cells, we hypothesized that the combination treatment would augment inhibition of the proinflammatory loop between CCR6^+^ memTh cells and RASF when compared with either compound alone. Similar to the effects of 1,25(OH)_2_D_3_ and DEX on CCR6^+^ memTh cell cultures, treatment of the CCR6^+^ memTh-RASF cocultures significantly reduced IL-17A, IL-22, and IFNγ production, especially with 1,25(OH)_2_D_3_. However, the production of TNFα was stimulated upon treatment with DEX, whereas it was still inhibited with the combination treatment. Furthermore, IL-10 was significantly upregulated in response to DEX and the combination of DEX and 1,25(OH)_2_D_3_, but not 1,25(OH)_2_D_3_ alone (Fig. 2).

Despite the differences in modulation of T cell-derived cytokines by DEX and 1,25(OH)_2_D_3_, they equally inhibited the RASF-derived factors IL-6, IL-8, and PGE2. For IL-6 and IL-8 there was also a trend towards additive inhibition in response to the combination of 1,25(OH)_2_D_3_ and DEX. Interestingly, DEX treatment led to a significantly greater inhibition of the tissue-destructive enzymes MMP1 and MMP3 than 1,25(OH)_2_D_3_ treatment (Fig. 2). These data suggest that, although no additive effect of 1,25(OH)_2_D_3_ and DEX is found on individual cytokines, the two compounds cooperate to reduce the proinflammatory milieu at an inflammatory synovial site by each targeting different players in the inflammation. Furthermore, the increased production of IL-10 potentially mediates anti-inflammatory effects on other immune cells.

### DEX is a stronger inhibitor of proinflammatory factors from RASF than 1,25(OH)_2_D_3_

**Fig. 3:**
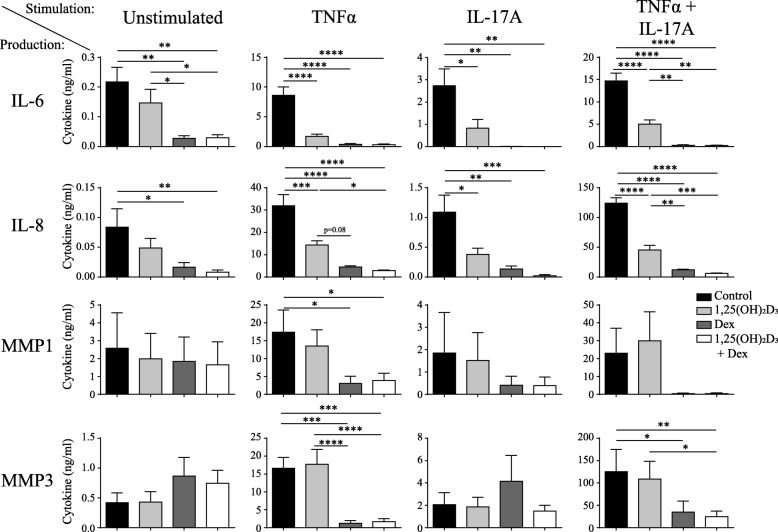
DEX is a more potent inhibitor of cytokine production by stimulated and unstimulated RASF. RASF were left unstimulated or were stimulated with tumor necrosis factor (TNF)α or interleukin (IL)-17A for 3 days and treated with or without 100 nM 1,25(OH)_2_D_3_ and 1000 nM dexamethasone (DEX). Production of IL-6, IL-8, matrix metalloprotease (MMP)1 and MMP3 were measured after 3 days in the culture supernatant using ELISA. Mean ± SEM are given for *n* = 6 RASF. *p < 0.05, **p < 0.01, ***p < 0.001, ****p < 0.0001

The finding that DEX is an equal inhibitor of IL-6 and IL-8 and stronger inhibitor of MMP1 and MMP3 than 1,25(OH)_2_D_3_ while being less efficient in inhibiting T cell-derived cytokines in the CCR6^+^ memTh-RASF cocultures suggests a direct effect of DEX on RASF. To investigate this, RASF were cultured with or without stimulation of TNFα, IL-17A, or both for 3 days and treated with 1,25(OH)_2_D_3_, DEX, or a combination (Fig. 3). Without stimulation, 1,25(OH)_2_D_3_ did not significantly affect cytokine production by RASF, whereas DEX reduced the levels of IL-6 and IL-8. Upon stimulation with TNFα, both 1,25(OH)_2_D_3_ and DEX inhibited IL-6 and IL-8, whereas MMP1 and MMP3 were only significantly inhibited by DEX or the combination treatment. Similar patterns were observed with stimulation using IL-17A or a combination of IL-17A and TNFα. Together with our previous findings, these data suggest that, although DEX and 1,25(OH)_2_D_3_ can affect both T cells and RASF, DEX acts most strongly on RASF whereas the main effect of 1,25(OH)_2_D_3_ is mediated via the T cells.

### Low dose of 1,25(OH)_2_D_3_ and DEX improves the effects of TNFα blockade in CCR6^+^ memTh-RASF cocultures

**Fig. 4:**
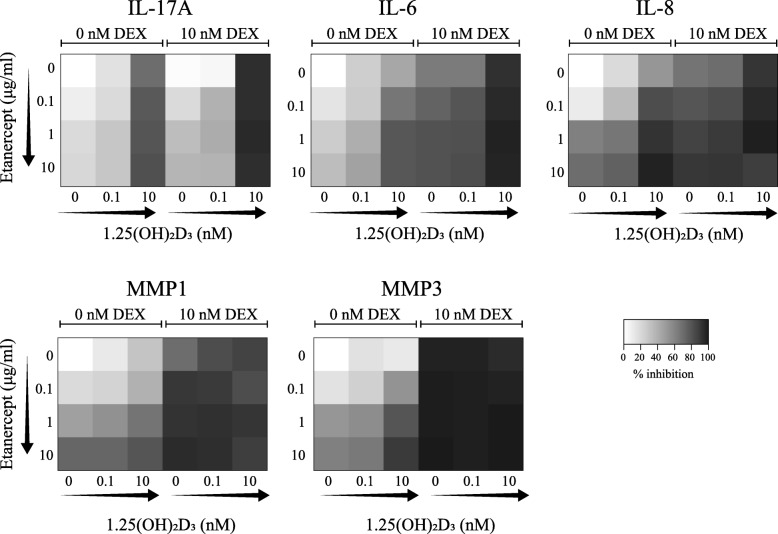
Suppression of RASF activation by TNFα blockade is enhanced by adding DEX and 1,25(OH)_2_D_3_. CCR6^+^ memTh-RASF cocultures as described in Fig. 2 were exposed to 10 nM dexamethasone (DEX), 0.1 or 10 nM 1,25(OH)_2_D_3_, and 0, 0.1, 1 or 10 μg/ml etanercept or combinations of the compounds as indicated. After 3 days, synovial fibroblast activation was measured through cytokine detection using ELISA. Arrows indicate increasing concentrations. Heatmaps are constructed using the mean inhibition in each condition of *n* = 6 healthy donors cultured on RASF from two different RA patients. IL interleukin, MMP matrix metalloprotease

Since 1,25(OH)_2_D_3_ and DEX additively inhibit the proinflammatory loop between CCR6^+^ memTh cells and RASF, we next studied whether the 1,25(OH)_2_D_3_-DEX combination can enhance the anti-inflammatory effect of TNFα blockade in this model system. To make these experiments more physiologically relevant, we used a dose-testing experiment to determine whether physiologically relevant dosages of 1,25(OH)_2_D_3_ and DEX still affected cytokine production in the CCR6^+^ memTh-RASF coculture system; 10 nM DEX still inhibited IL-6, IL-8, and MMP1 in CCR6^+^ memTh-RASF cocultures, but 0.1 nM 1,25(OH)_2_D_3_ only slightly affected these proinflammatory factors and did not appear different from the 1 nM dose (Additional file 1: Figure S2). Therefore, the value of adding DEX and 1,25(OH)_2_D_3_ to etanercept was assessed using 10 nM DEX and 0.1 or 10 nM 1,25(OH)_2_D_3_. CCR6^+^ memTh cells were sorted from healthy controls and cultured together with RASF while being exposed to various combinations of 1,25(OH)_2_D_3_, DEX, and the TNFα-blocking agent etanercept. Inhibition of IL-17A, IL-6, IL-8, MMP1, and MMP3 is shown as heatmaps in Fig. 4 and further detailed in Additional file 1 (Figure S3).

For IL-17A, etanercept dose-dependently inhibited cytokine expression, but this effect was stronger (darker shades) when the cells were also treated with 10 nM 1,25(OH)_2_D_3_. Combining DEX with this dose of 1,25(OH)_2_D_3_ further suppresses IL-17A and leaves little added effect for etanercept. IL-6 and IL-8 are more strongly inhibited by etanercept than IL-17A and show a trend towards a dose response (left column of the heatmap, top to bottom). Adding increasing dosages of 1,25(OH)_2_D_3_ augments the effects of etanercept, but the optimal effect is reached when 10 nM DEX, 10 nM 1,25(OH)_2_D_3_, and etanercept are combined. Notably, under these conditions there is no significant difference between 0.1 or 10 μg/ml etanercept and cytokine expression is more than 90% reduced. MMP1 and MMP3 show a similar pattern of inhibition as IL-6 and IL-8, except that the effect of DEX is stronger even without additional 1,25(OH)_2_D_3_ or etanercept. These data suggest that combining 1,25(OH)_2_D_3_ and especially DEX with etanercept has additive effects compared with etanercept alone.

**Fig. 5:**
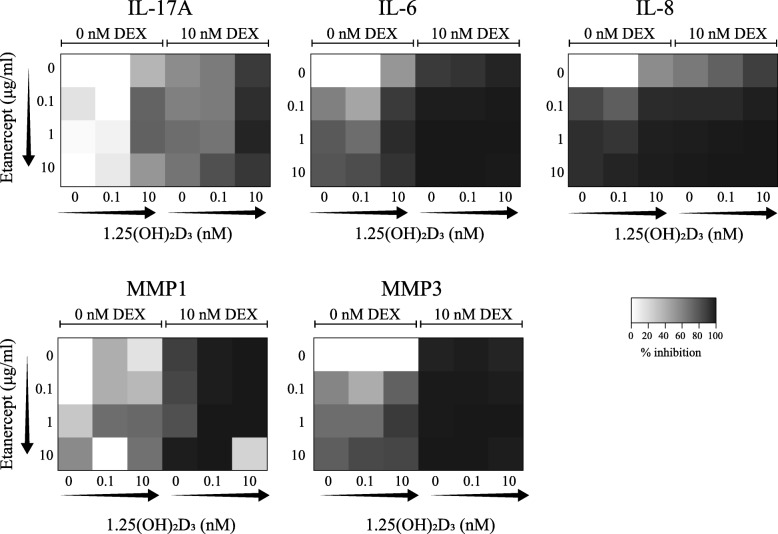
Additive effects of dexamethasone (DEX) and 1,25(OH)_2_D_3_ on the inhibition of RASF activation by TNFα blockade are verified for treatment-naive RA patients. Cocultures were set up as described in Fig. 4 using RASF from established RA patients and allogeneic CCR6^+^ memTh cells sorted from PBMCs of treatment-naive early RA patients. Cytokine expression was measured using ELISA after 3 days of culture. Arrows indicate increasing concentrations and heatmaps represent the mean inhibition per condition for *n* = 2–4 treatment-naive early RA patients. IL interleukin, MMP matrix metalloprotease

Since the cells from the healthy controls that were used in Fig. 4 and Additional file 1 (Figure S3) may react differently to treatment than the cells from RA patients, the experiment was repeated using sorted CCR6^+^ memTh cells from treatment-naive early RA patients cultured with RASF serving as a proof of principle (Fig. 5 and Additional file 1: Figure S4). Due to large variation between patients (Additional file 1: Figure S4), there was a less clear dose-dependent inhibition of IL-17A in response to etanercept. However, 10 nM 1,25(OH)_2_D_3_ still enhanced the effects of etanercept, and IL-17A inhibition was again further increased when 10 nM DEX was added. The inhibition of IL-6 and IL-8 by etanercept was stronger in the CCR6^+^ memTh cells from RA patients than those from healthy individuals. Similar to the results for the healthy controls, both 1,25(OH)_2_D_3_ and DEX enhanced the effects of etanercept. MMP1 and MMP3 were also inhibited by etanercept and their expression was further suppressed by DEX (Fig. 5).

Altogether, the data from Figs. 4 and 5 show that combination with DEX, and to a lesser extent 1,25(OH)_2_D_3_, provide a beneficial effect over TNFα blockade alone in a model for synovial inflammation.

## Discussion

This study showed that 1,25(OH)_2_D_3_ and DEX can additively inhibit synovial inflammation modeled by CCR6^+^ memTh-RASF cocultures. Furthermore, combining low doses of DEX and 1,25(OH)_2_D_3_ with TNFα blockade demonstrated added value over TNFα blockade alone.

Similar to our previous results in PBMCs and memTh cells [11], 1,25(OH)_2_D_3_ was a stronger modulator of IL-17A, IL-22, IFNγ, and IL-10 in CCR6^+^ memTh cells than DEX. This could be due to the reported resistance of CCR6^+^ memTh cells to cytokine inhibition and apoptosis induction by GCs [13]. Interestingly, in asthma, GC resistance is increased with decreasing vitamin D serum levels [14]. Since the vitamin D receptor can enhance the activity of the GC receptor at promoter sites, 1,25(OH)_2_D_3_ may be able to overcome GC resistance through this mechanism [15]. Notably, in Crohn’s disease Th17.1 cells, one of the subpopulations within CCR6^+^ memTh cells, are the most GC-resistant cells [16]. Since these cells can also be found at the site of inflammation in juvenile idiopathic arthritis [17] and RA (unpublished observations) further research into cell type-specific modulation by GCs may further elucidate its immunosuppressive actions in RA.

Independent of the differences in modulation of cytokines derived from CCR6^+^ memTh cells, either cultured alone or together with RASF, both 1,25(OH)_2_D_3_ and DEX inhibited the activation of synovial fibroblasts as demonstrated by decreased IL-6, IL-8, MMP1, MMP3, and PGE2. These data suggest that DEX has strong direct effects on RASF, which was confirmed by RASF-only cultures. Although others have shown that 1,25(OH)_2_D_3_ can inhibit MMP1 and MMP3 from RASF under IL-1β stimulation [18], this was not observed in our cultures with stimulation of TNFα and IL-17A. Interestingly, whereas TNFα stimulation of RASF was generally more potent than stimulation with IL-17A, the combination of TNFα and IL-17A induced a higher level of IL-6 and MMP1 and a striking stronger increase in IL-8 and MMP3. It has been previously observed in keratinocytes that IL-8 was more strongly induced by TNFα and IL-17A than IL-6 [19]. Also, another study in RA fibroblast-like synoviocytes suggested a slightly stronger additive effect on IL-8 than IL-6, although they only used 1 ng/ml for both TNFα and IL-17A [20]. Since DEX and 1,25(OH)_2_D_3_ inhibit IL-8 and MMP3 both after TNFα single stimulation or TNFα-IL-17A combination, this may be an effective way to suppress even the strong stimulation that is potentially present in the RA joint.

Based on other studies showing that the vitamin D receptor and GC receptor can cooperate to enhance one another’s functions [15, 21], we also expected synergistic effects of 1,25(OH)_2_D_3_ and DEX in CCR6^+^ memTh cells or RASF. However, this synergy was not observed, except that TNFα could only be inhibited when 1,25(OH)_2_D_3_ and DEX were combined. Instead, the data indicate that 1,25(OH)_2_D_3_ and DEX additively suppress inflammation through targeting different inflammatory pathways. 1,25(OH)_2_D_3_ indirectly reduces RASF activation through modulation of IL-17A and could reduce activation of other immune cells, such as macrophages, by inhibiting IFNγ [22]. DEX, on the other hand, directly affects the RASF and reduces immune cell activation, immune cell attraction, and tissue destruction through regulation of IL-6, IL-8, and MMPs, respectively. Finally, 1,25(OH)_2_D_3_ and DEX are both capable of inducing the anti-inflammatory cytokine IL-10 in CCR6^+^ memTh cell monocultures or in coculture with RASF, which could further contribute to inhibiting synovial inflammation.

Because of the strong immunomodulatory effects of 1,25(OH)_2_D_3_ and DEX, we postulated that they could be beneficial in the treatment of autoimmune diseases. This study demonstrated that DEX especially could augment the effect of TNFα blockade on RASF activation, even up to the point that no differences could be seen between the lowest and highest dose of anti-TNFα. Adding 0.1 nM 1,25(OH)_2_D_3_ did not contribute to this effect, but 10 nM enhanced the effects of DEX and TNFα blockade. The concentration of 0.1 nM (approximately 40 pg/ml) corresponds to the 20–80 pg/ml of 1,25(OH)_2_D_3_ that has been found in the synovial fluid of RA patients [23]. However, the synovial fluid is not always a perfect representation of the situation in the synovium. Furthermore, it has been reported that immune cells are capable of converting 25(OH)D_3_ into 1,25(OH)_2_D_3_ [24, 25], suggesting that the local concentration of active vitamin D in the inflamed synovium may be higher than 0.1 nM and therefore could contribute to the anti-inflammatory effects of DEX and anti-TNFα.

Although these data indicate that a combination of DEX and TNFα blockade could be beneficial in the treatment of RA, some limitations of this study should be considered. Firstly, the current study has focused on an in-vitro culture model with only T cells and RASF, whereas an inflamed joint also contains other cell types [26]. Although 1,25(OH)_2_D_3_ and DEX are both known for their wide range of immunomodulatory properties [12, 27], the exact effects of combining these with TNFα blockade have not been investigated. Furthermore, the data suggest that dose reduction of TNFα blockade could be possible on combination with DEX since there is no dose-dependent effect anymore. It should be noted that the physiological concentration of etanercept in the synovial fluid has not been elaborately studied. One study found the concentration of etanercept in the synovial fluid was around 20 ng/ml in two patients receiving 50 mg etanercept every 2 weeks after 5 weeks, thus in between two dosages [28]. However, one patient who received 50 mg every week had a concentration of 100 ng/ml after 5 weeks, suggesting that the concentration shortly after etanercept injection is in the range of the 0.1 μg/ml that was used in this study [28]. The inhibitory effects of RASF activation by this dose of etanercept have been drastically enhanced by DEX and 1,25(OH)_2_D_3_.

A final point to consider is that TNFα blockade has more effects than only suppressing RASF activation [29]. Therefore, it is possible that our in-vitro model overlooks interactions that may arise between the compounds and a more complex environment. To study this, murine models for RA, such as collagen-induced arthritis, could be used as a first validation of our study. After that, a clinical trial should be designed in which a low dose of DEX is combined with TNFα blockade. Due to the perceived immunomodulatory effects of 1,25(OH)_2_D_3_, adding vitamin D supplements to this treatment will not only prevent the osteoporotic side effects of DEX, but it may also further enhance the therapeutic effects.

## Conclusion

This study suggests an added value of combining 1,25(OH)_2_D_3_ with DEX for inhibiting synovial inflammation in RA patients through their distinct cell type-specific modulation properties. Furthermore, the findings in the CCR6^+^ memTh-RASF functional coculture model provide a rationale for a clinical trial combining low-dose 1,25(OH)_2_D_3_ and DEX with TNFα blockade to improve disease management in RA.

## Additional file

Additional file 1: **Table S1.** Characteristics of patients used in this study. **Figure S1.** 1,25(OH)_2_D_3_ and DEX minimally affect apoptosis in CCR6^+^ memTh cells. **Figure S2.** Low doses of 1,25(OH)_2_D_3_ and DEX still modulate RASF activation by CCR6^+^ memTh cells. **Figure S3.** Complete data behind the heatmaps of Fig. 4. **Figure S4.** Complete data behind the heatmaps of Fig. 5. (DOCX 2328 kb)

## Abbreviations

CCR: C-C chemokine receptor
DEX: Dexamethasone
DMARD: Disease-modifying antirheumatic drug
GC: Glucocorticoid
IFN: Interferon
IL: Interleukin
memTh: Memory T helper
MMP: Matrix metalloprotease
PBMC: Peripheral blood mononuclear cell
PGE2: Prostaglandin E2
RA: Rheumatoid arthritis
RASF: Rheumatoid arthritis synovial fibroblasts
RORC: RAR-related orphan receptor C
TNF: Tumor necrosis factor
