# Supplementary material for: 1,25(OH)2D3 and dexamethasone additively suppress synovial fibroblast activation by CCR6+ T helper memory cells and enhance the effect of tumor necrosis factor alpha blockade
Source: Arthritis Res Ther. 2018 Sep 20;20:212. doi: 10.1186/s13075-018-1706-9 (PMC6148958; doi:10.1186/s13075-018-1706-9)
Supplement: Supplementary file 1 — Table S1. Characteristics of patients used in this study. Figure S1. 1,25(OH)2D3 and DEX minimally affect apoptosis in CCR6+ memTh cells. Figure S2. Low doses of 1,25(OH)2D3 and DEX still modulate RASF activation by CCR6+ memTh cells. Figure S3. Complete data behind the heatmaps of Fig. 4. Figure S4. Complete data behind the heatmaps of Fig. 5. (DOCX 2328 kb) [file 13075_2018_1706_MOESM1_ESM.docx]

| **Parameters** | **n=4** |
| --- | --- |
| Age (years) | 66.0 (11) |
| Female, *n (%)* | 3 (75) |
| RF positive, *n (%)* | 3 (75) |
| ACPA positive, *n (%)* | 3 (75) |
| DAS | 5.1 (0.6) |
| VAS global | 63.8 (36.0) |
| ESR (mm/hr) | 42 (29) |
| CRP | 22 (17) |
| TJC44 | 19.8 (10.8) |
| SJC44 | 17.5 (7.0) |
| Disease duration (days) | 155 (90) |

**Table S1** Characteristics of patients used in this study.

Data are represented as mean (SD) unless otherwise indicated. Abbreviations: DAS, disease activity score for 44 joints; VAS, visual analogue scale; ESR, erythrocyte sedimentation rate; CRP, c-reactive protein; TJC44, tender joint count of 44 joints; SJC44, swollen joint count of 44 joints.

**
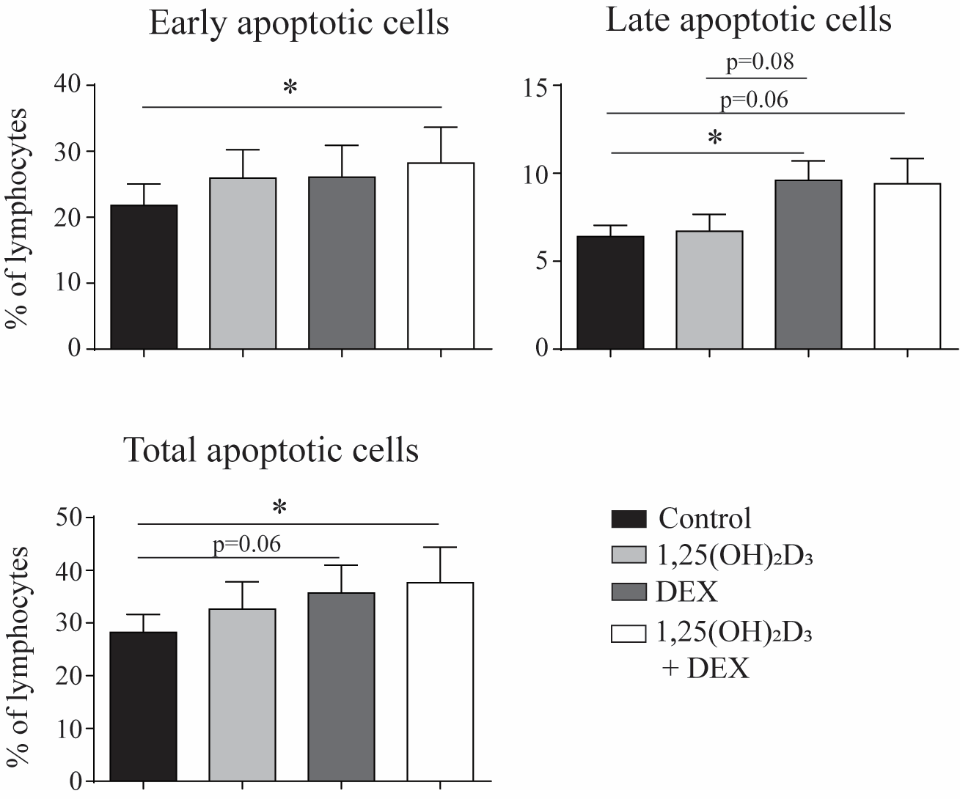
**

**Figure S1. 1,25(OH)_2_D_3_ and DEX minimally affect apoptosis in CCR6+ memTh cells.** Sorted CCR6+ memTh cells were cultured for three days with or without 100 nM 1,25(OH)_2_D_3_ and 1000 nM DEX under stimulation of anti-CD3 and anti-CD28. On day 3, apoptosis was measured using 7AAD-AnnexinV staining. Early apoptosis is defined as AnnexinV+7AAD-, late apoptotic cells are AnnexinV+7AAD+ and the total percentage apoptotic cells is depicting all AnnexinV+ cells. The data show mean ± SEM of n=5 healthy controls, representative of 2 independent experiments. *p<0.05

**
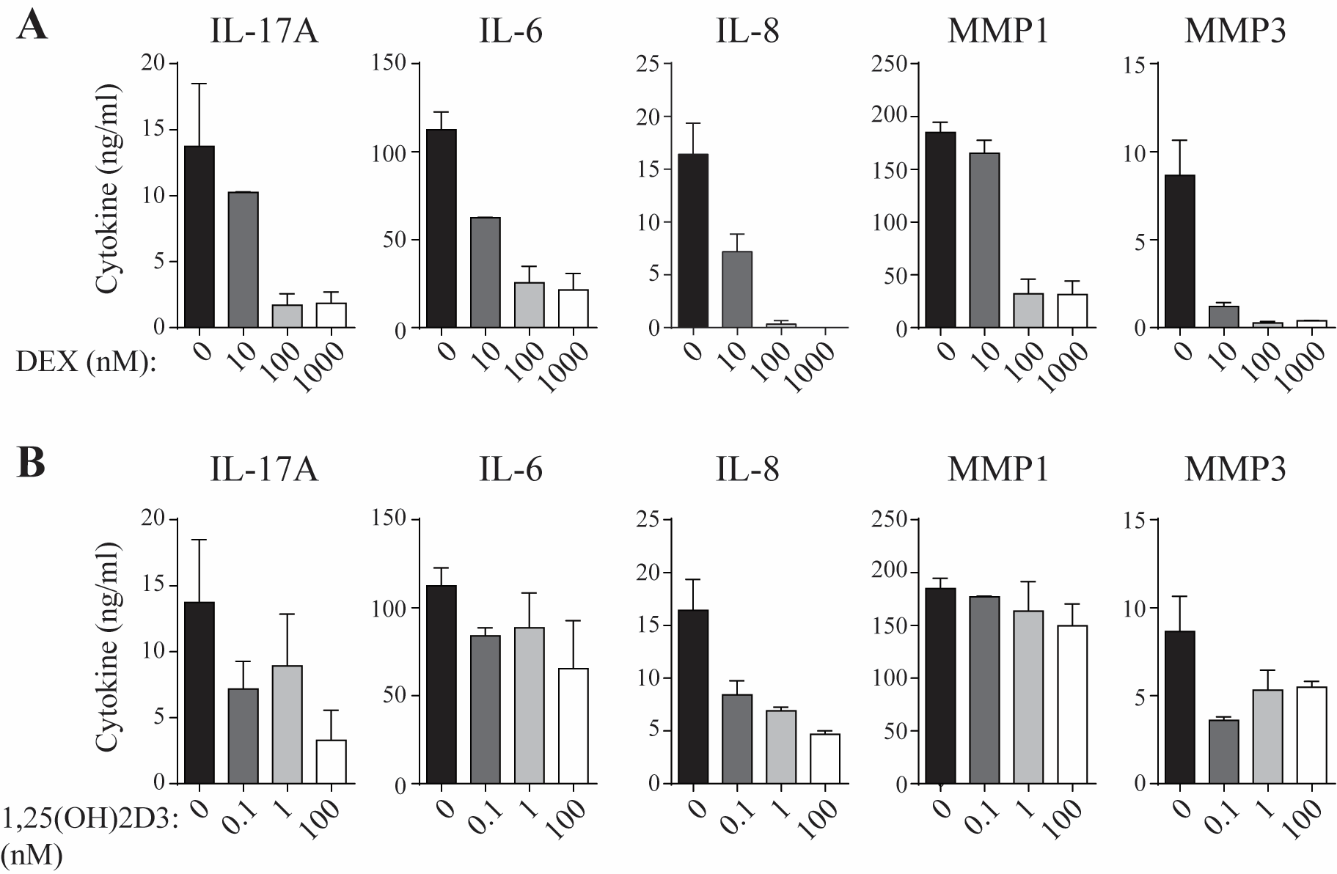
**

**Figure S2. Low doses of 1,25(OH)_2_D_3_ and DEX still modulate RASF activation by CCR6+ memTh cells.** Co-cultures of healthy CCR6+ memTh cells and RASF were treated with various concentrations of 1,25(OH)_2_D_3_ or DEX, while the cells were stimulated with anti-CD3 and anti-CD28. After three days cytokine production was assessed using ELISA. Results represent n=2-3 healthy donors and show mean ± SEM.

**
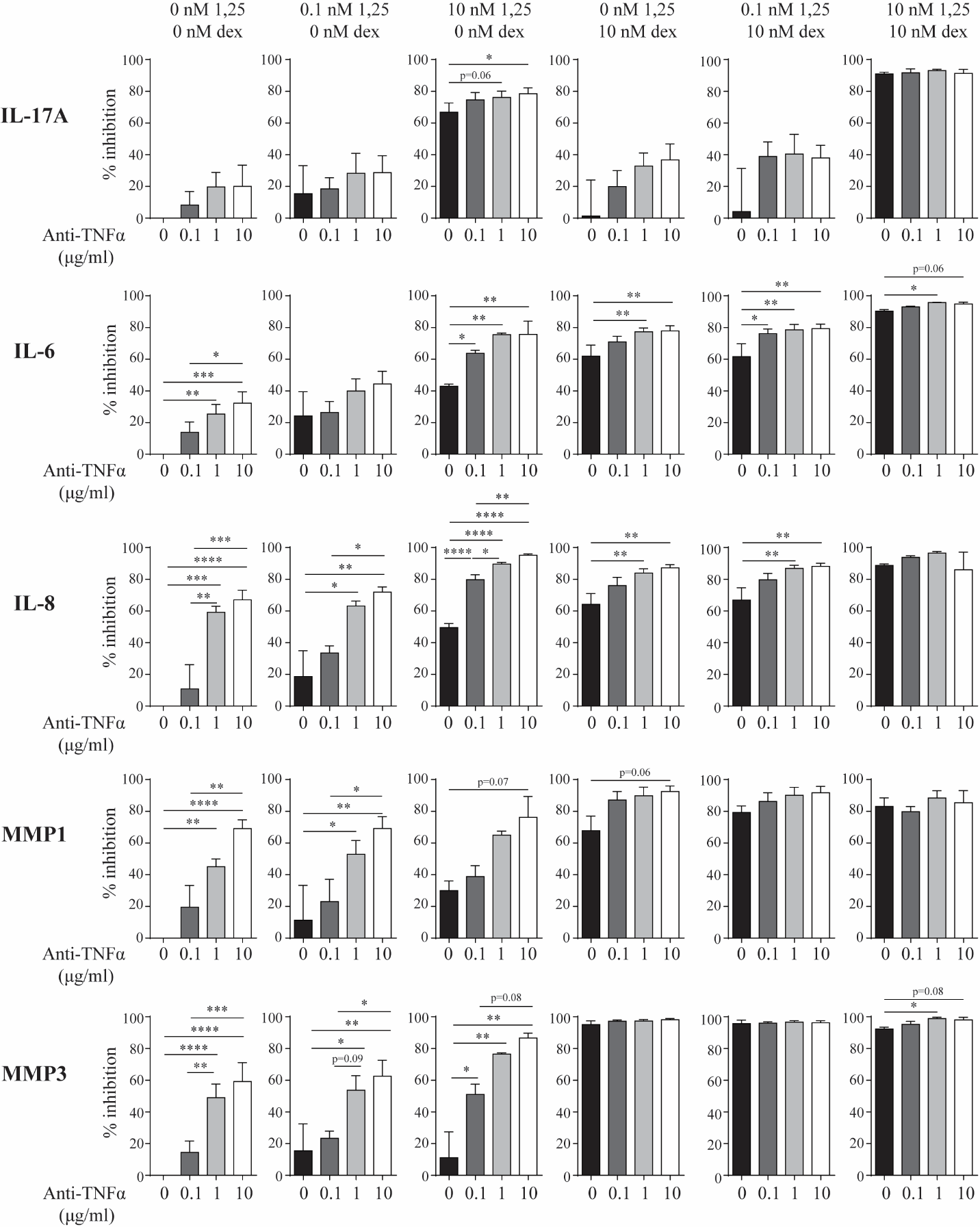
**

**Figure S3. Complete data behind the heatmaps of figure 4.** CCR6+ mTh-RASF co-cultures as described for figure 2 were exposed to 10 nM DEX, 0.1 or 10 nM 1,25(OH)_2_D_3_, 0, 0.1, 1 or 10 μg/ml etanercept (anti-TNFα) or combinations of the compounds as indicated. After three days, synovial fibroblast activation was measured through cytokine detection using ELISA. Data represent mean ± SEM for n=6 healthy donors on RASF from 2 different RA patients. Statistical tests were performed to compare the effects of etanercept dose using the given dose of DEX and 1,25(OH)_2_D_3_. *p<0.05, **p<0.01, ***p<0.001, ****p<0.0001

**
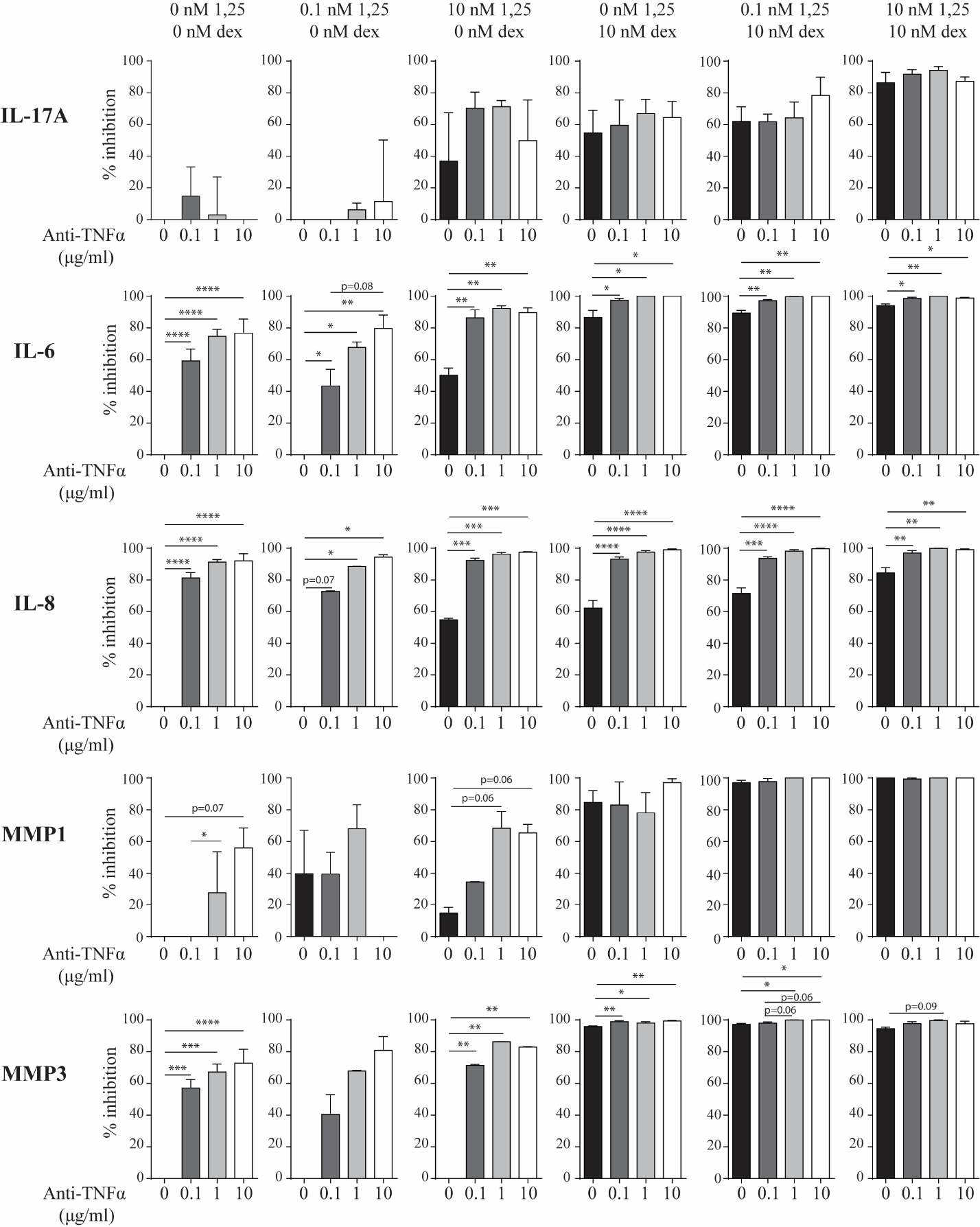
**

**Figure S4. Complete data behind the heatmaps of figure 5.** Co-cultures were set up as described for figure 4 using RASF from established RA patients and allogeneic CCR6+ mTh cells sorted from PBMC of treatment-naïve early RA patients. Cytokine expression was measured using ELISA after three days of culture. The graphs show mean ± SEM for n=2-4 treatment-naïve early RA patients. Statistical tests were performed to compare the effects of etanercept dose using the given dose of DEX and 1,25(OH)_2_D_3_. *p<0.05, **p<0.01, ***p<0.001, ****p<0.0001
